# Time-to-pregnancy and risk of cardiovascular disease among men and women

**DOI:** 10.1007/s10654-021-00718-8

**Published:** 2021-01-25

**Authors:** Maria C. Magnus, Abigail Fraser, Janet W. Rich-Edwards, Per Magnus, Deborah A. Lawlor, Siri E. Håberg

**Affiliations:** 1 Centre for Fertility and Health, Norwegian Institute of Public Health, P.O. Box 222, 0213 Skøyen, Oslo, Norway; 2 MRC Integrative Epidemiology Unit, University of Bristol, Bristol, UK; 3 Population Health Sciences, Bristol Medical School, Bristol, UK; 4 Bristol NIHR Biomedical Research Centre, Bristol, UK; 5 Division of Women’s Health, Department of Medicine, Brigham and Women’s Hospital, Boston, USA; 6 Department of Epidemiology, T.H. Chan School of Public Health, Harvard University, Boston, USA

**Keywords:** Time-to-pregnancy, Subfertility, Cardiovascular disease, MoBa

## Abstract

A few studies indicate that women with prolonged time-to-pregnancy (TTP) have an increased risk of cardiovascular disease (CVD). This has not been studied in men. We evaluated CVD risk by self-reported TTP among parous women (n = 64,064) and men (n = 50,533) participating in the Norwegian Mother, Father and Child Cohort Study. TTP was categorized as 0–3 (reference), 4–12 and > 12 months. CVD diagnosed between 2008 and 2017 were available from the national patient and general practitioner databases. Risk of CVD by TTP was estimated using Cox regression adjusting for baseline age, education, BMI, smoking, diabetes, and number of offspring in both sexes, and history of endometriosis, ovarian cysts, preterm birth and pre-eclampsia for women. Mean age was 33 for women and 35 for men at baseline (years). The rate of any CVD was 24 per 1000 person years among women and 22 per 1000 person years among men. Longer TTP was associated with increased rate of CVD among women, with adjusted hazard ratios (HRs) of 1.07 (95% CI: 1.03, 1.09) for TTP 4–12 months and 1.14 (1.08, 1.20) for TTP > 12 months. Among men, respective HRs for CVD were 1.06 (1.00, 1.10) for TTP 4–12 months and 1.07 (1.01, 1.14) for TTP > 12 months. We observed sex-differences in the relationship with CVD subtypes but none were statistically significant. In conclusion, both men and women with a prolonged TTP had a small increased risk of CVD, clinical significance of which is unclear. Further studies are necessary to investigate in detail what underlying causes of prolonged TTP might be reflected in the increased risk of CVD. Longer follow-up is required to confirm these preliminary findings.

**Supplementary information:**

The online version contains supplementary material available at (10.1007/s10654-021-00718-8).

## Background

Between 10 and 15% of couples try for more than 12 months before they conceive, which is often used as a cut off to define subfertility [1, 2]. Subfertility may be an early marker of chronic diseases risk, including the risk of cardiovascular disease (CVD) [3]. Two previous studies examined prolonged time-to-pregnancy (TTP) and risk of CVD in women [4, 5], including a small (N = 744) retrospective study and a Swedish registry-based study (N = 863,324), both supporting an increased risk of CVD with prolonged TTP in women. In contrast to these two studies, a Danish registry-based study (n = 87,221) found no increased risk of CVD among women who were diagnosed with fertility problems or seeking fertility treatment [6]. None of these studies evaluated the relationship among men.

Any increased risk of CVD among people with subfertility might have various potential explanations. Underlying explanations that might be shared between the sexes include sociodemographics (such age and education), psychosocial stress and other health related factors [such as smoking and body-mass index (BMI)] linked to subfertility and known to influence the risk of CVD [7–10]. Other sex-specific factors that might be associated with differences in CVD risk according to subfertility include sex-hormone levels, underlying disorders related to fertility potential, use of assisted reproductive technologies and pregnancy complications [11–15]. Specific disorders that contribute to subfertility and an increased risk of CVD include polycystic ovarian syndrome (PCOS) [16, 17] and endometriosis [18] in women, and varicoceles in men [19–21]. The large proportion of unexplained subfertility (20–30%) [1, 2], and the underdiagnosis of conditions contributing to fertility problems [22–24], highlight the value of looking further at the relationship between TTP and risk of CVD.

The objective of the current study was to examine the relationship between TTP and CVD in both men and women. This will determine if subfertile couples should be followed more closely for future CVD risk. The purpose of this study was not to disentangle what factor(s) related to subfertility might increase CVD risk, but to understand whether subfertility may be a marker of future CVD risk in young adults who may benefit from earlier efforts of CVD detection and prevention.

## Methods

### The Norwegian mother, father and child cohort study

We studied women and men participating in the Norwegian Mother, Father and Child Cohort Study (MoBa) [25]. MoBa recruited pregnant women (about 95,000) and their partners (about 75,000) across Norway between 1999 and 2009. A written informed consent was obtained from all participants. The participation rate of pregnant women was 41%. Information from participants was obtained through questionnaires at the time of recruitment and at regular follow-up intervals. We obtained information on deliveries from the birth registry and CVD events from the Norwegian patient registry (information available from 2008 onwards) and general practitioner data base (information available from 2006 onwards) [26]. For men and women participating in MoBa to be included in our analysis, we required that they had participated in the cohort with at least one planned pregnancy, had provided information on the TTP for at least one of these planned pregnancy, and that they were without reported CVD at baseline. Participants could have had other pregnancies that were not included in the cohort for which we did not know the TTP. We used self-reported information in MoBa questionnaires and combined these with registrations in the patient and general practitioner database made prior to baseline to identify prevalent CVD. Cases of CVD in the patient and general practitioner databases were only available from 2006 and 2008, respectively. Baseline was defined as January 1st 2008 or the time of recruitment for those recruited after this time, since this was when the national patient registry was established. The Norwegian data inspectorate approved the data collection in MoBa. The current study was approved by the Regional Committee for Medical and Health Research Ethics of South/East Norway.

### Time-to-pregnancy

At the time of recruitment, women were asked whether their current pregnancy was planned or not. If the pregnancy was planned, women were asked to indicate how many months they had regular intercourse without contraception before they became pregnant. The answer options were less than 1 month, 1–2 months, and 3 months or more. If they had been trying for more than 3 months, they indicated the exact number of months. Based on this information, we categorized TTP as 0–3 months, 4–12 months and more than 12 months. Pregnancies conceived by assisted reproductive technologies were given a TTP of more than 12 months. Women could participate in the cohort with more than one pregnancy, and the TTP was reported for each pregnancy included in the cohort. We therefore used the longest reported TTP as the exposure for women with more than one pregnancy. Men were not asked separately about TTP. The TTP reported by the women was assigned to their partners.

### Cardiovascular disease

We used self-reported information in MoBa questionnaires and registrations in the patient and general practitioner database prior to baseline to identify prevalent CVD. Notably, cases of CVD in the patient and general practitioner databases were only available from 2006 and 2008, respectively. At recruitment, participants self-reported hypertension, heart disease or other cardiovascular diseases. Incident cases were identified through the registries. Diagnoses in the patient registry are coded according to the international classification of diseases version 10 (ICD-10), while diagnoses in general practitioner database are coded according to the international classification of primary care version 2 (ICPC-2). The main outcome was overall CVD. We also separately examined hypertensive disorders, ischemic heart disease, cerebrovascular disease, atrial fibrillation/flutter, atherosclerosis and other CVD disorders. See supplementary material “Appendix 1” for details on the administrative codes used to define the CVD outcomes. We had follow-up information on CVD available from the registries until December 31st, 2017. Validations studies that have been conducted on some of the more common diagnosis codes indicated a high accuracy of the registrations in the health registries [27–29].

### Covariates

From the medical birth registry, information was available on age (continuous) and number of children (0, 1, 2, and 3 or higher). We obtained self-reported information through questionnaires on educational level (less than high school, high school, up to four years of university and more than 4 years of university), smoking status (never, former and current), body-mass index (weight in kg/height in m^2^), and diabetes mellitus (yes versus no). For women, we also explored the role of pregnancy complications in previous pregnancies as recorded in the birth registry, including any history of a preterm birth (defined as < 37 gestational weeks) or pre-eclampsia in a prior pregnancy, and self-reported history of endometriosis (yes vs. no) and ovarian cysts (yes vs. no) obtained through questionnaires. These background characteristics were obtained at recruitment (in the middle of the index pregnancy). We therefore assume that the men and women did not substantially change their lifestyle, finish an ongoing education, or get a new diagnosis between when they started trying to conceive and the middle of the index pregnancy.

### Statistical analysis

We described the risk of CVD according to TTP categories using Kaplan Meier plots. We examined the magnitude of the association between TTP and risk of CVD using Cox proportional hazards regression. Women and men free of CVD at baseline were followed from January 1st 2008 (or the date of recruitment for those recruited after this time) until their first registration of CVD, death from other causes or December 31st 2017 for those who were alive and not diagnosed with CVD. To evaluate the proportional hazards assumption, we examined the Schoenfeld residuals. In multivariable analysis, we adjusted for age, education, BMI, smoking, diabetes and number of children. We further adjusted for history of preterm birth, pre-eclampsia, endometriosis and ovarian cysts for women. Prolonged TTP is influenced by female fertility problems, male fertility problems or a combination of the two. Couples also share a lifestyle which might influence their fertility. One important example is partners’ correlated BMI [30]. We therefore adjusted for partners characteristics as the exposure is a couple level exposure, and shared behaviours may be associated with own CVD risk. The main analyses examined overall CVD risk, while secondary analyses describe the risk of CVD subtypes. Missing information on covariates were imputed by chained equations. This multiple imputation approach assumes missing at random. Using a non-parametric bootstrapping test, we compared the associations in men and women. We conducted sensitivity analyses excluding couples who had conceived by assisted reproductive technologies. We also conducted a sensitivity analysis where we included men and women who did not plan their pregnancy in the reference category of TTP 0–3 months. We further evaluated the role of pregnancy planning by conducting a stratified analysis according to use of contraceptives in the 12 months prior to conception.

As TTP is a couple level exposure, it is difficult to distinguish whether female, male or joint factors contribute to a prolonged TTP. As we are considering TTP as a proxy for subfertility, a large number of women and men may be misclassified (i.e. they are not subfertile but their partner is). We therefore conducted sensitivity analyses randomly assigning 20, 40, 60 and 80 percent of individuals in the categories of prolonged TTP to be in the reference category (TTP 0–3 months). We did this random reassignment 1000 times using bootstrapping.

All analyses were conducted using Stata version 15 (Statacorp, Texas).

## Results

**Fig. 1:**
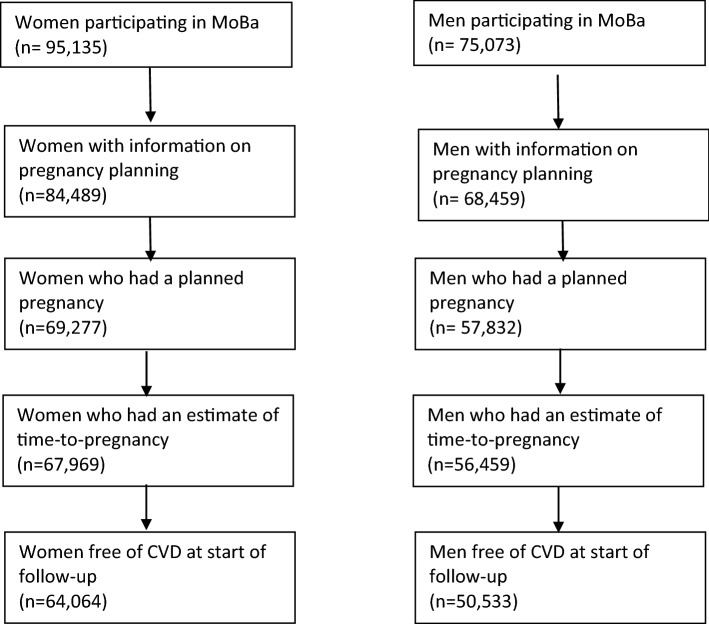
Illustration of the eligible study population

MoBa includes 95,135 women and 75,073 men. After restricting to those with at least one planned pregnancy (about 80%) in MoBa, with information on TTP and who were alive and free of CVD at baseline (90% of men and 95% of women), 64,064 women and 50,533 men were included in analyses (Fig. 1). The number of couples available is the same as the number of men (n = 50,533). The greater number of women compared to men/couples is due to the fact that a lot of women participated in the cohort without their partner. No men participated in the cohort without their partner. About 60% of couples conceived within 3 months, 27% between 4–12 months, and 12% after more than 12 months. The distribution of background characteristics is presented in Table 1. The average follow-up time was 8.9 years (range 0.1–10.5 years). The age range at the end of follow-up was between 27 and 62 for women (mean 43), and between 28 and 78 for men (mean 45).

**Table 1:** Distribution of background characteristics among eligible men and women without any history of cardiovascular disease at baseline

Characteristics	Women (n = 64,064)	Men (n = 50,533)
Age, mean(SD)	33.2 (4.7)	35.2 (5.3)
*Education, N(%)*		
Less than high school	4096 (6.4)	4106 (8.1)
High school	17,927 (28.0)	18,821 (37.2)
Up to 4 years of college	26,663 (41.6)	14,034 (27.8)
More than 4 years of college	15,118 (23.6)	12,192 (24.1)
Missing	260 (0.4)	1380 (2.7)
BMI, mean(SD)	23.9 (4.0)	25.8 (3.2)
Missing, N(%)	1589 (2.5)	1753 (3.5)
*Smoking, N(%)*		
Never	33,097 (51.7)	24,443 (48.4)
Former	16,982 (26.5)	11,256 (22.3)
Current	13,710 (21.4)	14,436 (28.6)
Missing	275 (0.4)	398 (0.8)
*Self-reported diabetes mellitus, N(%)*		
No	63,709 (99.5)	50,145 (99.2)
Yes	355 (0.6)	388 (0.8)
*Parity, N(%)*		
0	29,616 (46.2)	24,586 (48.7)
1	23,219 (36.2)	17,726 (35.1)
2	9099 (14.2)	6737 (13.3)
3 +	2130 (3.3)	1484 (2.9)
*Endometriosis*		
No	62,990 (98.3)	NA
Yes	1074 (1.7)	NA
*Ovarian cysts*		
No	60,280 (94.1)	NA
Yes	3784 (5.9)	NA
*History of preterm birth, N(%)*		
No	61,169 (95.5)	NA
Yes	2895 (4.5)	NA
*History of pre-eclampsia, N(%)*		
No	62,179 (97.1)	NA
Yes	1885 (2.9)	NA
*Time-to-pregnancy, N(%)*		
0–3	38,691 (60.4)	30,979 (61.3)
4–12	17,510 (27.3)	13,518 (26.8)
More than 12	7863 (12.3)	6036 (11.9)
*Use contraceptive in the past year, N(%)*		
No	22,401 (35.0)	17,191 (34.0)
Yes	41,663 (65.0)	33,342 (66.0)

**Fig. 2:**
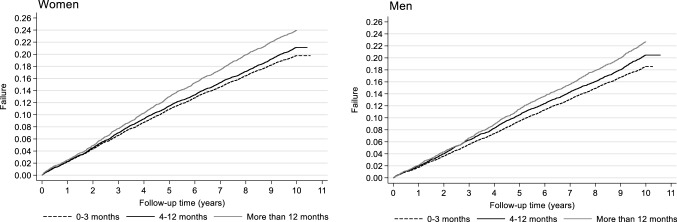
Kaplan Meier curves showing the risk of cardiovascular disease across time according to the categories of time-to-pregnancy

**Fig. 3:**
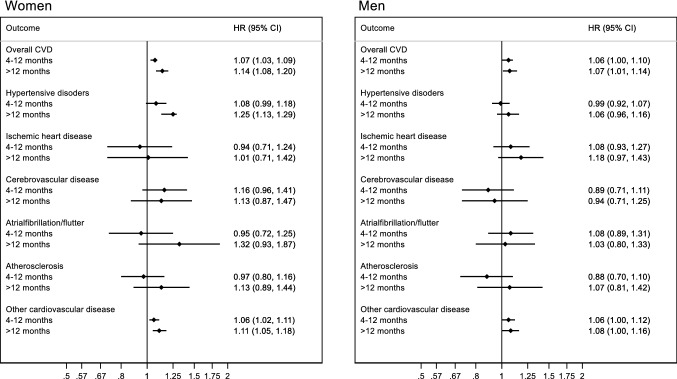
Risk of cardiovascular disease according to time-to-pregnancy among women (n = 65,584) and men (m = 51,039). The reference group are those with a time-to-pregnancy of 3 months or less. Adjusted for age, education, smoking status, body-mass index, diabetes mellitus and number of offspring

The risk of overall CVD was 24 per 1000 person years for women and 22 per 1000 person years for men. The Kaplan Meier curve indicated a modest increased risk of CVD among women with prolonged TTP (Fig. 2). Similar observations were made in men (Fig. 2). For overall CVD, the adjusted hazard ratio (HR) was 1.07 (95% CI: 1.03, 1.09) for women with a TTP of 4–12 months and 1.14 (95% CI: 1.08, 1.20) for women with a TTP of more than 12 months, compared to women who conceived within 0–3 months (Fig. 3; supplementary material “Appendix 2”). The results were slightly attenuated among women after adjustment for pregnancy complications and underlying disorders (supplementary material “Appendix 2”). The adjusted HR for overall CVD was 1.06 (95% CI: 1.00, 1.10) among men with a TTP 4–12 months and 1.07 (95% CI: 1.01, 1.14) among men with a TTP of more than 12 months (Fig. 3; supplementary material “Appendix 3”).

The risk of hypertensive disorders was increased both among women with TTP 4–12 (adjusted HR 1.08; 95% CI: 0.99, 1.18) and TTP more than 12 months (adjusted HR 1.25; 95% CI: 1.13, 1.29), compared to TTP 0–3 months (Fig. 3; supplementary material “Appendix 2”). There was no increased risk of hypertensive disorders associated with TTP among men (Fig. 3; supplementary material “Appendix 3”). In women, there was also a tendency for an incresed risk of cerebrovascular disease, with an adjusted HR of 1.16 (95% CI: 0.96, 1.41) with a TTP of 4–12 months and 1.13 (95% CI: 0.87, 1.47) with a TTP of more than 12 months (Fig. 3; supplementary material “Appendix 2”). In men, there was weak evidence of an increased risk of ischemic heart disease with TTP more than 12 months, with an adjusted HR of 1.18 (95% CI: 0.97, 1.43) (Fig. 3; supplementary material “Appendix 3”). There was no statistical evidence of differences in the associations between the sexes from the bootstrapping test (*p* values > 0.2). The only exception was the estimated association between TTP 4–12 months and risk of cerebrovascular disease (*p* value 0.05). The results were similar after excluding men and women who used assisted reproductive technologies. The results also did not change after additional adjustment for characteristics of the partner (supplementary material appendices 2 and 3).

We excluded women and men with unplanned index pregnancies from the main analysis as they lacked a reliable TTP. Individuals with unplanned pregnancies were younger, had a lower education, were more likely to smoke and were more likely to be first-time parents (supplementary material “Appendix 4”). Women but not men with unplanned pregnancies had an increased CVD risk (supplementary material “Appendix 5”). As a sensitivity analysis, we included individuals with unplanned pregnancies in the category with a TTP of 0–3 months, yielding similar results to the main analysis (supplementary material appendices 6 and 7). The relationship between TTP and CVD was similar among those who had and had not used contraceptives in the past year, the small numbers yielded imprecise estimates (supplementary material appendices 8 and 9). Based on information provided by women, we compared background characteristics in men (their respective partners) according to participation in the cohort. We found that men who participated in the cohort were younger, had a higher educational attainment, were less likely to smoke and more likely to have planned their pregnancy compared to men who did not participate (supplementary material “Appendix 10”). Restricting the evaluation to CVD diagnosed in the specialist health-care services also yielded similar findings (supplementary material appendices 11 and 12).

Our sensitivity analyses randomly reassigning a proportion of individuals in the categories of prolonged TTP to be in the reference group resulted in an attenuation of our findings (supplementary material “Appendix 13”). The attenuation of the findings increased with the increasing proportion of individuals who were reassigned (from 20 to 80%).

## Discussion

We observed an increased risk of overall CVD with prolonged TTP among both men and women. The magnitude of the relationship appeared greater among women, but this needs to be further explored in future studies. There were some notable differences in the relationship between TTP and CVD subgroups between the sexes. For example, women with a prolonged TTP had an increased risk of cerebrovascular disease, while men with a prolonged TTP had an increased risk of ischemic heart disease. Despite the large study population, there was no strong statistical support for differences between the sexes. However, in this relatively young population, it is important to note that for both sexes the majority of overall CVD events were ‘other’ category (disorders other than hypertensive disorders, ischemic heart disease, cerebrovascular disease, atrial fibrillation/flutter or atherosclerosis). As this is the first examination of TTP and CVD risk in both sexes, our findings require replication. We note that TTP is a complex couple-level measure likely to reflect a broad range of underlying factors. We were not able to disentangle what specific aspects related to subfertility might be reflected in the increased risk of CVD with the information available in MoBa.

Strengths of our study include the sample size, our evaluation of men, the detailed information on potential confounders, and information on TTP for several pregnancies to increase the likelihood of capturing lifetime history of subfertility. This allowed us to evaluate CVD subtypes and to estimate the highest reported TTP for men and women who participated with more than one pregnancy. Information on most confounders was obtained through questionnaires, and some misclassification is expected.

Our study had has some limitations. The participation rate in MoBa was 41%. Selection into the cohort is influenced by age, number of offspring, underlying diseases and other characteristics [31]. MoBa also only includes couples who conceived, and we could not evaluate the risk of CVD among men and women who did not conceive after trying for a long time. Both of these selection factors could have led us to underestimate the association between prolonged TTP and risk of CVD, if couples with more severe subfertility who did not conceive have a higher risk of CVD than those who conceive. Information on background characteristics was obtained at recruitment reflecting the status at the time of pregnancy. Therefore it is possible that these characteristics changed between the time that the couple started trying to conceive and they became pregnant. Notably, age-adjusted estimates were generally similar to multivariable estimates. The patient (registration from 2008) and general (registration from 2006) practitioner databases did not cover the whole recruitment period of MoBa, and we may have missed some incident cases between recruitment and when the follow-up from the registries started. We only had follow-up information available from the registries through December 2017, which limited our power particularly for the analysis of CVD subgroups. The participants in this study were still relatively young at the end of follow-up. CVD at such a young age could be indicative of genetic abnormalities, and the subtypes of CVD that present at such a young age are more likely to be influenced by genetics [32]. It is also possible that infertility is caused by or indicates such underlying genetic abnormalities, and our results might therefore be influenced by genetic confounding. This could have led us to overestimate the association of interest. We used women´s report of TTP and assigned this to their partners. This could have introduced misclassification. However, as TTP is a couple-level measure of fertility problems, it is unlikely that we would have obtained a more accurate answer if we had asked the men directly. Any discrepancy in the longest reported TTP for partners participating in MoBa would have reflected a switch of partners.

Since we used TTP as a proxy for subfertility, and TTP is a couple level measure, we conducted a sensitivity analyses where we randomly reassigned a proportion of individuals in the two categories of prolonged TTP to be in the reference group. We did this because individuals may be assigned to prolonged TTP because of their partners’ subfertility. These sensitivity analyses indicated that any individual misclassification due to the fact that TTP is a couple-level measure resulted in a modest overestimation of the associations. This challenge in the interpretation of associations of TTP with CVD has not been adequately discussed in previous papers.

Our study is in line with previous studies reporting an increased risk of CVD among women with prolonged TTP. A cross-sectional study of 744 U.S. women age 20–59 reported a 1.83 times higher odds (95% CI: 1.15, 2.89) of heart failure, coronary heart disease, heart attack or stroke among women with a TTP > 12 months [4]. A Swedish registry-based study indicated an increased risk of coronary heart disease, stroke or heart failure among women who reported having tried for more than 5 years to conceived compared to those who conceived within the first year, with an adjusted HR of 1.19 (95% CI: 1.02, 1.39), while there was no increased risk among women who reported having tried for 1–2 (HR 1.07; 95% CI: 0.92, 1.23) or 3–4 years (HR 1.01; 0.83, 1.23) [5]. The findings from these two studies contrast results from a Danish registry-based study reporting no increased risk of ischemic heart disease or cerebrovascular disease among women diagnosed with infertility, adjusted hazard ratio 0.98 (95% CI: 0.85, 1.14) [6]. The heterogeneity in the findings across studies is likely influenced by differences in the definition of subfertility or prolonged TTP, the CVD outcomes examined, and the age composition of the study populations.

Previous studies have not examined the relationship between prolonged TTP and risk of CVD in men. An increased risk of CVD is reported among both childless men and women [33–35]. However, the interpretation of these studies is difficult since childlessness can be voluntary. Our finding of a small increased CVD risk among men with prolonged TTP, as well as the possible greater risk among women than men would benefit from replication.

There may be differences in the relationships between TTP and CVD subtypes between the sexes, although our findings were inconclusive due to modest number of cases. Specifically, TTP was associated with a modest increased risk of cerebrovascular disease only in women, while an increased risk of ischemic heart disease according to prolonged TTP was only seen in men. CVD is more likely to first manifest as cerebrovascular disease in women and ischemic heart disease in men [36]. Based on what we know about the risk of CVD subtypes according to known CVD risk factors, cerebrovascular is more closely linked to hypertension while ischemic heart disease is more linked to hypercholesterolemia [37]. Our observation that the risk of hypertensive disorders associated with TTP was only robustly evident among women and not men therefore corresponds well with an increased risk of cerebrovascular disease. Due to our limited follow-up time and the relatively young age of the participants at the end of follow-up, these relationships need to be further investigated.

A prolonged TTP is associated with greater risk of pregnancy complications [38, 39], and several pregnancy complications are associated with increased risk of CVD among women [12]. Whether use of assisted reproductive technologies influences the risk of CVD in women remains unclear [13, 14]. Interestingly, accounting for women’s history of pregnancy complications, endometriosis, ovarian cysts and use of assisted reproductive technologies only slightly attenuated our findings. These findings highlight that there might be other aspects related to subfertility among women that warrant further investigation.

In conclusion, women and men with a prolonged TTP may have an increased risk of CVD. Our findings indicate that there may be differences in the risk of CVD subtypes associated with prolonged TTP between the sexes, although this needs to be further investigated. Further studies are also necessary to investigate in detail what underlying causes of prolonged TTP might be reflected in the increased risk of CVD.

## Supplementary information

Supplementary file1 (DOCX 304kb)

## Data Availability

Data are available by contacting the Norwegian Mother, Father and Child Cohort Study administration (datatilgang@fhi.no).
