# Supplementary material for: Time-to-pregnancy and risk of cardiovascular disease among men and women
Source: Eur J Epidemiol. 2021 Jan 25;36(4):383–91. doi: 10.1007/s10654-021-00718-8 (PMC8076115; doi:10.1007/s10654-021-00718-8)
Supplement: Supplementary file 1 — Supplementary file1 (DOCX 304kb) [file 10654_2021_718_MOESM1_ESM.docx]

Appendix 1. Administrative codes used to define cardiovascular disease in the health registries

| Outcome | Patient registry  ICD-10 codes | General practitioner database  ICPC-2 codes |
| --- | --- | --- |
| Overall cardiovascular disease | I00-I99 | K70, K71, K74-K80 K82-K84, K86-K88, K90-K96 |
| Hypertensive disorders | I10-15 | K86-87 |
| Ischemic heart disease | I20-I25 | K74-76 |
| Cerebrovascular disease | I60-69 | K90-91 |
| Atrial fibrillation/flutter | I48 | K78 |
| Atherosclerosis | I70 | K92 |
| Other | I00-09, I26-47, I49-52, I71-99 | K70-71, K77, K79-84, K88, K93-96 |

| Outcome | Time-to-pregnancy | Person time in years | Events | Age-adjusted  HR (95% CI) | Adjusted  ^a^  HR (95% CI) | Adjusted  ^b^  HR (95% CI) | Adjusted  ^c^  HR (95% CI) |
| --- | --- | --- | --- | --- | --- | --- | --- |
| Overall CVD | 0-3 months | 334,105 | 9,241 | Ref | Ref | Ref | Ref |
|  | 4-12 months | 149,830 | 4,521 | 1.08 (1.04, 1.12) | 1.07 (1.03, 1.09) | 1.07 (1.03, 1.11) | 1.08 (1.03, 1.12) |
|  | More than 12 months | 66,180 | 2,209 | 1.17 (1.12, 1.23) | 1.14 (1.08, 1.20) | 1.12 (1.07, 1.18) | 1.10 (1.03, 1.16) |
| Hypertensive disorders | 0-3 months | 375,407 | 1,612 | Ref | Ref | Ref | Ref |
|  | 4-12 months | 169,395 | 867 | 1.14 (1.05, 1.24) | 1.08 (0.99, 1.18) | 1.08 (0.99, 1.18) | 1.11 (1.01, 1.23) |
|  | More than 12 months | 75,363 | 531 | 1.43 (1.29, 1.57) | 1.25 (1.13, 1.39) | 1.24 (1.11, 1.37) | 1.20 (1.06, 1.37) |
| Ischemic heart disease | 0-3 months | 381,988 | 164 | Ref | Ref | Ref | Ref |
|  | 4-12 months | 172,988 | 76 | 0.94 (0.71, 1.23) | 0.94 (0.71, 1.24) | 0.93 (0.71, 1.23) | 0.95 (0.67, 1.35) |
|  | More than 12 months | 77,591 | 45 | 1.04 (0.74, 1.44) | 1.01 (0.71, 1.42) | 0.95 (0.66, 1.35) | 0.92 (0.58, 1.45) |
| Cerebrovascular disease | 0-3 months | 381,168 | 311 | Ref | Ref | Ref | Ref |
|  | 4-12 months | 172,601 | 164 | 1.15 (0.95, 1.39) | 1.16 (0.96, 1.41) | 1.16 (0.95, 1.40) | 1.26 (1.00, 1.58) |
|  | More than 12 months | 77,465 | 74 | 1.11 (0.86, 1.43) | 1.13 (0.87, 1.47) | 1.11 (0.85, 1.45) | 1.03 (0.74, 1.44) |
| Atrialfibrillation/flutter | 0-3 months | 381,904 | 170 | Ref | Ref | Ref | Ref |
|  | 4-12 months | 173,003 | 73 | 0.94 (0.71, 1.24) | 0.95 (0.72, 1.25) | 0.95 (0.72, 1.25) | 1.04 (0.76, 1.42) |
|  | More than 12 months | 77,609 | 46 | 1.29 (0.93, 1.80) | 1.32 (0.93, 1.87) | 1.31 (0.92, 1.87) | 1.15 (0.74, 1.79) |
| Atherosclerosis | 0-3 months | 380,656 | 399 | Ref | Ref | Ref | Ref |
|  | 4-12 months | 172,560 | 168 | 0.92 (0.77, 1.10) | 0.97 (0.80, 1.16) | 0.96 (0.80, 1.16) | 0.97 (0.78, 1.20) |
|  | More than 12 months | 77,393 | 89 | 1.05 (0.84, 1.33) | 1.13 (0.89, 1.44) | 1.11 (0.87, 1.42) | 1.08 (0.81, 1.43) |
| Other cardiovascular diseases | 0-3 months | 342,635 | 7,576 | Ref | Ref | Ref | Ref |
|  | 4-12 months | 154,272 | 3,640 | 1.06 (1.02, 1.11) | 1.06 (1.02, 1.11) | 1.06 (1.02, 1.11) | 1.07 (1.02, 1.12) |
|  | More than 12 months | 68,655 | 1,710 | 1.12 (1.06, 1.18) | 1.11 (1.05, 1.18) | 1.09 (1.04, 1.16) | 1.07 (1.00, 1.14) |

Appendix 2. Risk of cardiovascular disease according to time-to-pregnancy among women (n=64,064)

^a^ Adjusted for age, education, smoking status, body-mass index, diabetes mellitus and parity. ^b^ Adjusted for age, education, smoking status, body-mass index, diabetes mellitus, parity, history of preterm birth, history of pre-eclampsia, endometriosis and ovarian cysts. ^c^ Adjusted for own and partner age, education, smoking status, body-mass index, diabetes mellitus, parity, history of preterm birth, history of pre-eclampsia, endometriosis and ovarian cysts.

Appendix 3. Risk of cardiovascular disease according to time-to-pregnancy among men (n= 50,533)

| Outcome | Time-to-pregnancy | Person time in years | Events | Age-adjusted  HR (95% CI) | Adjusted ^a^  HR (95% CI) | Adjusted ^b^  HR (95% CI) |
| --- | --- | --- | --- | --- | --- | --- |
| Overall CVD | 0-3 months | 273,209 | 6,679 | Ref | Ref | Ref |
|  | 4-12 months | 117,929 | 3,166 | 1.08 (1.03, 1.12) | 1.05 (1.00, 1.10) | 1.05 (1.00, 1.10) |
|  | More than 12 months | 52,037 | 1,578 | 1.13 (1.07, 1.19) | 1.07 (1.01, 1.14) | 1.08 (1.02, 1.15) |
| Hypertensive disorders | 0-3 months | 295,203 | 2,342 | Ref | Ref | Ref |
|  | 4-12 months | 128,517 | 1,105 | 1.5 (0.98, 1.13) | 0.99 (0.92, 1.07) | 0.98 (0.91, 1.06) |
|  | More than 12 months | 56,883 | 621 | 1.19 (1.09, 1.30) | 1.06 (0.96, 1.16) | 1.05 (0.95, 1.16) |
| Ischemic heart disease | 0-3 months | 303,785 | 487 | Ref | Ref | Ref |
|  | 4-12 months | 132,461 | 255 | 1.15 (0.99, 1.34) | 1.08 (0.93, 1.27) | 1.08 (0.91, 1.27) |
|  | More than 12 months | 58,911 | 150 | 1.26 (1.04, 1.51) | 1.18 (0.97, 1.43) | 1.17 (0.96, 1.43) |
| Cerebrovascular disease | 0-3 months | 304,451 | 292 | Ref | Ref | Ref |
|  | 4-12 months | 133,023 | 120 | 0.91 (0.74, 1.12) | 0.89 (0.71, 1.11) | 0.88 (0.70, 1.11) |
|  | More than 12 months | 59,270 | 62 | 0.93 (0.71, 1.23) | 0.94 (0.71, 1.25) | 0.96 (0.72, 1.29) |
| Atrialfibrillation/flutter | 0-3 months | 304,592 | 337 | Ref | Ref | Ref |
|  | 4-12 months | 132,939 | 169 | 1.11 (0.93, 1.34) | 1.08 (0.89, 1.31) | 1.08 (0.89, 1.32) |
|  | More than 12 months | 59,347 | 82 | 1.08 (0.85, 1.38) | 1.03 (0.80, 1.33) | 1.05 (0.81, 1.36) |
| Atherosclerosis | 0-3 months | 305,131 | 278 | Ref | Ref | Ref |
|  | 4-12 months | 133,182 | 115 | 0.92 (0.74, 1.14) | 0.88 (0.70, 1.10) | 0.85 (0.67, 1.07) |
|  | More than 12 months | 59,451 | 68 | 1.10 (0.84, 1.44) | 1.07 (0.81, 1.42) | 1.09 (0.82, 1.45) |
| Other cardiovascular diseases | 0-3 months | 284,809 | 4,307 | Ref | Ref | Ref |
|  | 4-12 months | 123,557 | 2,033 | 1.07 (1.02, 1.13) | 1.06 (1.00, 1.12) | 1.07 (1.01, 1.13) |
|  | More than 12 months | 54,944 | 971 | 1.10 (1.02, 1.18) | 1.08 (1.00, 1.16) | 1.10 (1.02, 1.18) |

^a^ Adjusted for age, education, smoking status, body-mass index, diabetes mellitus and parity.

^b^ Adjusted for own and partner age, education, smoking status, body-mass index, diabetes mellitus and parity.

Appendix 4. Distribution of background characteristics among men and women according to pregnancy planning

| Characteristics | Women | | Men | |
| --- | --- | --- | --- | --- |
|  | Planned pregnancy  (n=69,277) | Unplanned pregnancy  (n=15,212) | Planned pregnancy  (n=57,832) | Unplanned pregnancy  (n=10,627) |
| **Age, mean(SD)** | 33.3 (4.7) | 31.9 (6.2) | 35.5 (5.4) | 34.2 (6.8) |
| **Education, N(%)** |  |  |  |  |
| Less than high school | 4,512 (6.5) | 2,578 (16.9) | 4,820 (8.3) | 1,632 (15.4) |
| High school | 19,453 (28.2) | 5,945 (39.1) | 21,554 (37.3) | 4,765 (44.8) |
| Up to 4 years of college | 28,611 (41.3) | 4,538 (29.8) | 15,980 (27.6) | 2,207 (20.8) |
| More than 4 years of college | 16,247 (23.5) | 2,028 (13.3) | 13,767 (23.8) | 1,565 (14.7) |
| Missing | 454 (0.7) | 123 (0.8) | 1,711 (3.0) | 458 (4.3) |
| **BMI, mean(SD)** | 24.0 (4.0) | 23.8 (4.3) | 25.9 (3.2) | 25.8 (3.5) |
| Missing | 1,939 (2.8) | 600 (3.9) | 2,178 (3.8) | 533 (5.1) |
| **Smoking, N(%)** |  |  |  |  |
| Never | 35,604 (51.4) | 6,074 (39.9) | 26,404 (45.7) | 4,235 (39.9) |
| Former | 18,398 (26.6) | 3,280 (21.6) | 12,271 (21.2) | 2,000 (18.8) |
| Current | 14,814 (21.4) | 5,800 (38.1) | 15,696 (27.1) | 4,302 (40.5) |
| Missing | 461 (0.4) | 58 (0.4) | 3,461 (6.0) | 90 (0.9) |
| **Self-reported diabetes mellitus, N(%)** |  |  |  |  |
| No | 68,691 (99.2) | 15,089 (99.2) | 54,302 (93.9) | 10,515 (98.9) |
| Yes | 421 (0.6) | 123 (0.8) | 489 (0.9) | 112 (1.1) |
| Missing | 165 (0.2) |  | 3,041 (5.3) | 0 (0) |
| **Parity, N(%)** |  |  |  |  |
| 0 | 31,562 (45.6) | 7,492 (49.3) | 27,414 (47.4) | 5,492 (51.7) |
| 1 | 24,903 (36.0) | 3,638 (23.9) | 20,386 (35.3) | 2,469 (23.2) |
| 2 | 9,847 (14.2) | 2,693 (17.7) | 7,759 (13.4) | 1,791 (16.9) |
| 3+ | 2,321 (3.4) | 1,210 (8.0) | 1,758 (3.0) | 755 (7.1) |
| Missing | 644 (0.9) | 179 (1.2) | 515 (0.9) | 120 (1.1) |
| **Cardiovascular disease at baseline, N(%)** |  |  |  |  |
| No | 66,344 (95.8) | 14,562 (95.7) | 54,438 (94.1) | 9,953 (93.7) |
| Yes | 2,933 (4.2) | 650 (4.3) | 3,394 (5.9) | 674 (6.3) |

Appendix 5. Kaplan Meier plot of risk of cardiovascular disease according to pregnancy planning among men and women

Women who did not plan their pregnancies had a modest increased risk of cardiovascular disease, with a hazard ratio of 1.08 (95% CI: 1.04, 1.13). For men, the HR for cardiovascular disease associated with unplanned pregnancy was 0.99 (95% CI: 0.95, 1.04).

Appendix 6. Risk of cardiovascular disease according to time-to-pregnancy among women including women with unplanned pregnancies in the reference category (n=79,980)

| Outcome | Time-to-pregnancy | Person time in years | Events | Age-adjusted  HR (95% CI) | Adjusted  ^a^  HR (95% CI) | Adjusted  ^b^  HR (95% CI) |
| --- | --- | --- | --- | --- | --- | --- |
| Overall CVD | 0-3 months | 453,527 | 12,915 | Ref | Ref | Ref |
|  | 4-12 months | 149,830 | 4,521 | 1.05 (1.01, 1.08) | 1.05 (1.02, 1.09) | 1.05 (1.02, 1.09) |
|  | More than 12 months | 66,180 | 2,209 | 1.13 (1.08, 1.18) | 1.12 (1.06, 1.17) | 1.10 (1.04, 1.15) |
| Hypertensive disorders | 0-3 months | 510,851 | 2,351 | Ref | Ref | Ref |
|  | 4-12 months | 169,395 | 867 | 1.06 (0.98, 1.14) | 1.04 (0.96, 1.13) | 1.04 (0.96, 1.13) |
|  | More than 12 months | 75,363 | 531 | 1.31 (1.20, 1.45) | 1.21 (1.09, 1.33) | 1.18 (1.07, 1.31) |
| Ischemic heart disease | 0-3 months | 520,112 | 259 | Ref | Ref | Ref |
|  | 4-12 months | 172,988 | 76 | 0.81 (0.63, 1.05) | 0.88 (0.68, 1.14) | 0.87 (0.67, 1.13) |
|  | More than 12 months | 77,591 | 45 | 0.90 (0.66, 1.24) | 0.95 (0.69, 1.32) | 0.89 (0.64, 1.25) |
| Cerebrovascular disease | 0-3 months | 519,046 | 464 | Ref | Ref | Ref |
|  | 4-12 months | 172,601 | 164 | 1.05 (0.87, 1.25) | 1.10 (0.92, 1.32) | 1.10 (0.92, 1.32) |
|  | More than 12 months | 77,465 | 74 | 1.02 (0.80, 1.31) | 1.08 (0.84, 1.39) | 1.07 (0.83, 1.38) |
| Atrialfibrillation/flutter | 0-3 months | 520,122 | 245 | Ref | Ref | Ref |
|  | 4-12 months | 173,003 | 73 | 0.89 (0.68, 1.15) | 0.91 (0.69, 1.18) | 0.91 (0.69, 1.18) |
|  | More than 12 months | 77,609 | 46 | 1.22 (0.89, 1.68) | 1.25 (0.90, 1.75) | 1.23 (0.88, 1.74) |
| Atherosclerosis | 0-3 months | 518,666 | 529 | Ref | Ref | Ref |
|  | 4-12 months | 172,560 | 168 | 0.94 (0.79, 1.12) | 0.99 (0.83, 1.18) | 0.99 (0.83, 1.18) |
|  | More than 12 months | 77,394 | 89 | 1.08 (0.86, 1.35) | 1.15 (0.91, 1.45) | 1.14 (0.90, 1.45) |
| Other cardiovascular diseases | 0-3 months | 465,593 | 10,522 | Ref | Ref | Ref |
|  | 4-12 months | 154,272 | 3,640 | 1.04 (1.00, 1.08) | 1.05 (1.01, 1.09) | 1.05 (1.01, 1.09) |
|  | More than 12 months | 68,655 | 1,710 | 1.09 (1.04, 1.15) | 1.10 (1.04, 1.16) | 1.08 (1.02, 1.14) |

^a^ Adjusted for age, education, smoking status, body-mass index, diabetes mellitus and parity.

^b^ Adjusted for age, education, smoking status, body-mass index, diabetes mellitus, parity, history of preterm birth, history of pre-eclampsia, endometriosis and ovarian cysts.

Appendix 7. Risk of cardiovascular disease according to time-to-pregnancy among men including men with unplanned pregnancies in the reference category (n= 60,873)

| Outcome | Time-to-pregnancy | Person time in years | Events | Age-adjusted  HR (95% CI) | Adjusted ^a^  HR (95% CI) |
| --- | --- | --- | --- | --- | --- |
| Overall CVD | 0-3 months | 358,291 | 8,854 | Ref | Ref |
|  | 4-12 months | 117,929 | 3,166 | 1.06 (1.02, 1.10) | 1.05 (1.00, 1.10) |
|  | More than 12 months | 52,037 | 1,578 | 1.11 (1.05, 1.17) | 1.07 (1.01, 1.14) |
| Hypertensive disorders | 0-3 months | 387,547 | 3,110 | Ref | Ref |
|  | 4-12 months | 128,517 | 1,105 | 1.04 (0.97, 1.11) | 0.99 (0.92, 1.07) |
|  | More than 12 months | 56,883 | 621 | 1.18 (1.08, 1.28) | 1.06 (0.96, 1.16) |
| Ischemic heart disease | 0-3 months | 398,715 | 685 | Ref | Ref |
|  | 4-12 months | 132,461 | 255 | 1.08 (0.93, 1.24) | 1.08 (0.93, 1.27) |
|  | More than 12 months | 58,911 | 150 | 1.18 (0.99, 1.41) | 1.18 (0.97, 1.43) |
| Cerebrovascular disease | 0-3 months | 399,694 | 403 | Ref | Ref |
|  | 4-12 months | 133,023 | 120 | 0.86 (0.70, 1.06) | 0.89 (0.71, 1.11) |
|  | More than 12 months | 59,270 | 62 | 0.90 (0.68, 1.17) | 0.94 (0.71, 1.25) |
| Atrial fibrillation/flutter | 0-3 months | 400,056 | 440 | Ref | Ref |
|  | 4-12 months | 132,939 | 169 | 1.12 (0.93, 1.33) | 1.08 (0.89, 1.31) |
|  | More than 12 months | 59,347 | 82 | 1.08 (0.85, 1.37) | 1.03 (0.80, 1.33) |
| Atherosclerosis | 0-3 months | 400,730 | 368 | Ref | Ref |
|  | 4-12 months | 133,182 | 115 | 0.91 (0.74, 1.13) | 0.88 (0.70, 1.10) |
|  | More than 12 months | 59,451 | 68 | 1.11 (0.86, 1.44) | 1.07 (0.81, 1.42) |
| Other cardiovascular diseases | 0-3 months | 373,967 | 5,669 | Ref | Ref |
|  | 4-12 months | 123,557 | 2,033 | 1.07 (1.01, 1.12) | 1.06 (1.00, 1.12) |
|  | More than 12 months | 54,944 | 971 | 1.09 (1.02, 1.17) | 1.08 (1.00, 1.16) |

^a^ Adjusted for age, education, smoking status, body-mass index, diabetes mellitus and parity.

| Appendix 8. Risk of cardiovascular disease according to time-to-pregnancy among women including women according to use of contraception in the past year (n=64,064) | | | | | | | | | |
| --- | --- | --- | --- | --- | --- | --- | --- | --- | --- |
|  |  | Used contraceptives in the past year | | | | Did not use contraceptives in the past year | | | |
| Outcome | Time-to-pregnancy | Person time in years | Events | Age-adjusted  HR (95% CI) | Adjusted  ^a^  HR (95% CI) | Person time in years | Events | Age-adjusted  HR (95% CI) | Adjusted  ^a^  HR (95% CI) |
| Overall CVD | 0-3 months | 265,465 | 7,326 | Ref | Ref | 68,640 | 1,915 | Ref | Ref |
|  | 4-12 months | 90,243 | 2,612 | 1.04 (1.00, 1.09) | 1.03 (0.99. 1.08) | 59,587 | 1,909 | 1.14 (1.07, 1.22) | 1.15 (1.07, 1.22) |
|  | More than 12 months | 3,563 | 128 | 1.27 (1.07, 1.51) | 1.23 (1.03, 1.47) | 62,617 | 2,081 | 1.17 (1.10, 1.25) | 1.15 (1.08, 1.23) |
| Hypertensive disorders | 0-3 months | 298,421 | 1,247 | Ref | Ref | 76,986 | 365 | Ref | Ref |
|  | 4-12 months | 101,665 | 485 | 1.11 (1.00, 1.23) | 1.06 (0.95, 1.18) | 67,730 | 382 | 1.17 (1.01, 1.35) | 1.11 (0.96, 1.29) |
|  | More than 12 months | 4,115 | 28 | 1.45 (0.99, 2.10) | 1.33 (0.92, 1.94) | 71,249 | 503 | 1.40 (1.22, 1.60) | 1.22 (1.06, 1.41) |
| Ischemic heart disease | 0-3 months | 303,513 | 121 | Ref | Ref | 78,475 | 43 | Ref | Ref |
|  | 4-12 months | 103,729 | 33 | 0.75 (0.51, 1.10) | 0.76 (0.52, 1.12) | 69,259 | 43 | 1.09 (0.72, 1.67) | 1.09 (0.71, 1.68) |
|  | More than 12 months | 4,192 | 6 | 2.89 (1.27, 6.57) | 2.96 (1.29, 6.77) | 73,399 | 39 | 0.86 (0.56, 1.33) | 0.85 (0.54, 1.34) |
| Cerebrovascular disease | 0-3 months | 302910 | 237 | Ref | Ref | 78,258 | 74 | Ref | Ref |
|  | 4-12 months | 103,404 | 108 | 1.32 (1.05, 1.65) | 1.31 (1.03, 1.65) | 69,197 | 56 | 0.85 (0.60, 1.21) | 0.92 (0.65, 1.32) |
|  | More than 12 months | 4,198 | 3 | 0.86 (0.28, 2.70) | 0.89 (0.28, 2.79) | 73,266 | 71 | 1.01 (0.73, 1.40) | 1.06 (0.75, 1.51) |
| Atrialfibrillation/flutter | 0-3 months | 303,403 | 132 | Ref | Ref | 78,500 | 38 | Ref | Ref |
|  | 4-12 months | 103,642 | 52 | 1.15 (0.84, 1.59) | 1.16 (0.84, 1.61) | 69,360 | 21 | 0.62 (0.36, 1.06) | 0.62 (0.36, 1.07) |
|  | More than 12 months | 4,202 | 4 | 2.19 (0.81, 5.92) | 2.30 (0.85, 6.27) | 73,407 | 42 | 1.13 (0.73, 1.76) | 1.21 (0.76, 1.94) |
| Atherosclerosis | 0-3 months | 302,426 | 312 | Ref | Ref | 78,230 | 87 | Ref | Ref |
|  | 4-12 months | 103,379 | 104 | 0.96 (0.77, 1.20) | 1.00 (0.80, 1.26) | 69,181 | 64 | 0.83 (0.60, 1.15) | 0.89 (0.64, 1.24) |
|  | More than 12 months | 4,185 | 5 | 1.11 (0.46, 1.27) | 1.22 (0.50, 2.95) | 73,208 | 84 | 1.02 (0.76, 1.38) | 1.12 (0.81, 1.54) |
| Other cardiovascular diseases | 0-3 months | 272,088 | 6,028 | Ref | Ref | 70,547 | 1,548 | Ref | Ref |
|  | 4-12 months | 92,798 | 2,105 | 1.02 (0.97, 1.07) | 1.02 (0.97, 1.07) | 61,474 | 1,535 | 1.14 (1.06, 1.22) | 1.15 (1.07, 1.24) |
|  | More than 12 months | 3,666 | 107 | 1.31 (1.08, 1.59) | 1.29 (1.06, 1.57) | 64,988 | 1,603 | 1.12 (1.04, 1.20) | 1.13 (1.05, 1.21) |

^a^ Adjusted for age, education, smoking status, body-mass index, diabetes mellitus and parity.

| Appendix 9. Risk of cardiovascular disease according to time-to-pregnancy among men according to use of contraception in the past year (n=50,533) | | | | | | | | | |
| --- | --- | --- | --- | --- | --- | --- | --- | --- | --- |
|  |  | Used contraceptives in the past year | | | | Did not use contraceptives in the past year | | | |
| Outcome | Time-to-pregnancy | Person time in years | Events | Age-adjusted  HR (95% CI) | Adjusted  ^a^  HR (95% CI) | Person time in years | Events | Age-adjusted  HR (95% CI) | Adjusted  ^a^  HR (95% CI) |
| Overall CVD | 0-3 months | 219,163 | 5,261 | Ref | Ref | 54,046 | 1,418 | Ref | Ref |
|  | 4-12 months | 71,550 | 1,906 | 1.09 (1.04, 1.15) | 1.07 (1.01, 1.13) | 46,379 | 1,260 | 1.04 (0.97, 1.12) | 1.00 (0.93, 1.09) |
|  | More than 12 months | 3,054 | 78 | 1.00 (0.80, 1.25) | 0.96 (0.76, 1.21) | 48,983 | 1,500 | 1.12 (1.04, 1.20) | 1.08 (1.00, 1.17) |
| Hypertensive disorders | 0-3 months | 236,651 | 1,806 | Ref | Ref | 58,552 | 536 | Ref | Ref |
|  | 4-12 months | 77,971 | 631 | 1.04 (0.95, 1.14) | 1.00 (0.91, 1.10) | 50,546 | 474 | 1.03 (0.91, 1.17) | 0.98 (0.86, 1.11) |
|  | More than 12 months | 3,306 | 32 | 1.15 (0.81, 1.63) | 1.07 (0.73, 1.55) | 53,577 | 589 | 1.13 (1.00, 1.27) | 1.04 (0.92, 1.18) |
| Ischemic heart disease | 0-3 months | 243,292 | 377 | Ref | Ref | 60,493 | 110 | Ref | Ref |
|  | 4-12 months | 80,163 | 152 | 1.20 (0.99, 1.44) | 1.15 (0.95, 1.40) | 52,298 | 103 | 1.09 (0.83, 1.43) | 1.01 (0.76, 1.33) |
|  | More than 12 months | 3,407 | 7 | 1.13 (0.54, 2.39) | 1.04 (0.46, 2.34) | 55,504 | 143 | 1.27 (0.99, 1.63) | 1.21 (0.93, 1.58) |
| Cerebrovascular disease | 0-3 months | 243,827 | 229 | Ref | Ref | 60,625 | 63 | Ref | Ref |
|  | 4-12 months | 80,523 | 65 | 0.84 (0.64, 1.11) | 0.84 (0.64, 1.12) | 52,500 | 55 | 1.01 (0.70, 1.45) | 0.98 (0.67, 1.44) |
|  | More than 12 months | 3,411 | 4 | 1.15 (0.43, 3.10) | 0.92 (0.29, 2.88) | 55,859 | 58 | 0.91 (0.64, 1.31) | 1.00 (0.69, 1.46) |
| Atrialfibrillation/flutter | 0-3 months | 244,058 | 252 | Ref | Ref | 60,535 | 85 | Ref | Ref |
|  | 4-12 months | 80,524 | 97 | 1.15 (0.91, 1.45) | 1.16 (0.92, 1.48) | 52,415 | 72 | 0.98 (0.72, 1.34) | 0.91 (0.65, 1.28) |
|  | More than 12 months | 3,407 | 6 | 1.58 (0.70, 3.55) | 0.83 (0.27, 2.61) | 55,940 | 76 | 0.90 (0.66, 1.22) | 0.87 (0.62, 1.21) |
| Atherosclerosis | 0-3 months | 244,381 | 224 | Ref | Ref | 60,751 | 54 | Ref | Ref |
|  | 4-12 months | 80,593 | 71 | 0.94 (0.72, 1.23) | 0.89 (0.67, 1.17) | 52,588 | 44 | 0.94 (0.63, 1.40) | 0.92 (0.61, 1.39) |
|  | More than 12 months | 3,405 | 4 | 1.19 (0.44, 3.20) | 1.22 (0.45, 3.28) | 56,046 | 64 | 1.20 (0.84, 1.73) | 1.14 (0.78, 1.67) |
| Other cardiovascular diseases | 0-3 months | 228,234 | 3,413 | Ref | Ref | 56,575 | 894 | Ref | Ref |
|  | 4-12 months | 74,772 | 1,261 | 1.12 (1.05, 1.19) | 1.11 (1.04, 1.19) | 48,785 | 772 | 1.00 (0.91, 1.10) | 0.98 (0.89, 1.08) |
|  | More than 12 months | 3,166 | 47 | 0.96 (0.72, 1.28) | 0.95 (0.71, 1.28) | 51,778 | 924 | 1.10 (1.00, 1.20) | 1.07 (0.97, 1.18) |

^a^ Adjusted for age, education, smoking status, body-mass index, diabetes mellitus and parity.

Appendix 10. Distribution of background characteristics among men who did and did not participate in the cohort based on information provided by women at recruitment

|  | **Men who did not participate**  **(n=20,062)** | **Men who did participate**  **(n=75,073)** |
| --- | --- | --- |
| **Age at recruitment, mean(SD)** | 33.1 (6.0) | 32.7 (5.4) |
| **Education, N(%)** |  |  |
| Less than high school | 2,646 (13.2) | 6,674 (8.9) |
| High school | 7,042 (35.1) | 26,859 (35.8) |
| Up to 4 years of college | 3,318 (16.5) | 18,388 (24.5) |
| More than 4 years of college | 2,440 (12.2) | 15,509 (20.7) |
| Missing | 4,616 (23.0) | 7,643 (10.2) |
| **BMI, mean(SD)** | 25.7 (3.2) | 25.8 (3.3) |
| Missing |  |  |
| **Smoking, N(%)** |  |  |
| Never/former | 11,243 (56.0) | 55,954 (74.5) |
| Current | 4,813 (24.0) | 13,245 (17.6) |
| Missing | 4,006 (20.0) | 5,874 (7.8) |
| **Pregnancy was planned, N(%)** |  |  |
| No | 11,658 (58.1) | 56,508 (75.3) |
| Yes | 4,402 (21.9) | 12,305 (16.4) |
| Missing | 4,002 (20.0) | 6,260 (8.3) |

Appendix 11. Risk of cardiovascular disease registered in the specialist health-care services according to time-to-pregnancy among women (n= 67,429)

| Outcome | Time-to-pregnancy | Person time in years | Events | Age-adjusted  HR (95% CI) | Adjusted  ^a^  HR (95% CI) | Adjusted  ^b^  HR (95% CI) |
| --- | --- | --- | --- | --- | --- | --- |
| Overall CVD | 0-3 months | 373,837 | 5,418 | Ref | Ref | Ref |
|  | 4-12 months | 169,016 | 2,595 | 1.04 (0.99, 1.09) | 1.04 (1.00, 1.10) | 1.04 (1.00, 1.10) |
|  | More than 12 months | 75,851 | 1,321 | 1.13 (1.07, 1.20) | 1.13 (1.06, 1.20) | 1.11 (1.04, 1.18) |
| Hypertensive disorders | 0-3 months | 380,676 | 455 | Ref | Ref | Ref |
|  | 4-12 months | 172,291 | 251 | 1.17 (1.00, 1.36) | 1.13 (0.96, 1.33) | 1.13 (0.96, 1.33) |
|  | More than 12 months | 77,051 | 167 | 1.58 (1.32, 1.89) | 1.37 (1.13, 1.66) | 1.34 (1.10, 1.62) |
| Ischemic heart disease | 0-3 months | 382,244 | 98 | Ref | Ref | Ref |
|  | 4-12 months | 173,082 | 51 | 1.04 (0.74, 1.45) | 1.06 (0.75, 1.49) | 1.05 (0.74, 1.49) |
|  | More than 12 months | 77,652 | 30 | 1.10 (0.73, 1.67) | 1.12 (0.73, 1.72) | 1.05 (0.68, 1.64) |
| Cerebrovascular disease | 0-3 months | 382,040 | 129 | Ref | Ref | Ref |
|  | 4-12 months | 173,025 | 71 | 1.15 (0.86, 1.54) | 1.18 (0.88, 1.58) | 1.17 (0.87, 1.58) |
|  | More than 12 months | 77,645 | 35 | 1.13 (0.77, 1.65) | 1.16 (0.79, 1.72) | 1.17 (0.79, 1.73) |
| Atrialfibrillation/flutter | 0-3 months | 382,036 | 138 | Ref | Ref | Ref |
|  | 4-12 months | 173,059 | 62 | 0.98 (0.72, 1.32) | 1.00 (0.74, 1.36) | 1.00 (0.74, 1.36) |
|  | More than 12 months | 77,664 | 32 | 1.08 (0.73, 1.60) | 1.10 (0.73, 1.65) | 1.07 (0.70, 1.63) |
| Atherosclerosis | 0-3 months | 382,439 | 39 | Ref | Ref | Ref |
|  | 4-12 months | 173,267 | 15 | 0.82 (0.45, 1.49) | 0.82 (0.44, 1.52) | 0.81 (0.44, 1.52) |
|  | More than 12 months | 77,754 | 8 | 0.89 (0.42, 1.93) | 0.95 (0.42, 2.10) | 0.89 (0.39, 2.00) |
| Other cardiovascular diseases | 0-3 months | 361,254 | 4,215 | Ref | Ref | Ref |
|  | 4-12 months | 163,478 | 1,986 | 1.03 (0.98, 1.09) | 1.04 (0.98, 1.09) | 1.03 (0.98, 1.09) |
|  | More than 12 months | 72,827 | 987 | 1.12 (1.04, 1.20) | 1.14 (1.06, 1.22) | 1.12 (1.04, 1.20) |

^a^ Adjusted for age, education, smoking status, body-mass index, diabetes mellitus and parity. ^b^ Adjusted for age, education, smoking status, body-mass index, diabetes mellitus, parity, history of preterm birth, history of pre-eclampsia, endometriosis and ovarian cysts.

Appendix 12. Risk of cardiovascular disease registered in the specialist health-care services according to time-to-pregnancy among men (n= 52,319)

| Outcome | Time-to-pregnancy | Person time in years | Events | Age-adjusted  HR (95% CI) | Adjusted ^a^  HR (95% CI) |
| --- | --- | --- | --- | --- | --- |
| Overall CVD | 0-3 months | 298,697 | 3,504 | Ref | Ref |
|  | 4-12 months | 130,090 | 1,703 | 1.09 (1.03, 1.15) | 1.05 (0.99, 1.12) |
|  | More than 12 months | 58,023 | 824 | 1.06 (0.99, 1.15) | 1.04 (0.96, 1.13) |
| Hypertensive disorders | 0-3 months | 312,341 | 836 | Ref | Ref |
|  | 4-12 months | 136,652 | 401 | 1.06 (0.94, 1.20) | 0.99 (0.88, 1.13) |
|  | More than 12 months | 61082 | 230 | 1.17 (1.01, 1.36) | 1.09 (0.93, 1.27) |
| Ischemic heart disease | 0-3 months | 314,358 | 403 | Ref | Ref |
|  | 4-12 months | 137,478 | 211 | 1.14 (0.96, 1.34) | 1.07 (0.90, 1.27) |
|  | More than 12 months | 61,706 | 116 | 1.09 (0.88, 1.34) | 1.03 (0.82, 1.28) |
| Cerebrovascular disease | 0-3 months | 315,251 | 176 | Ref | Ref |
|  | 4-12 months | 138,066 | 71 | 0.88 (0.67, 1.17) | 0.86 (0.65, 1.15) |
|  | More than 12 months | 61,989 | 46 | 1.08 (0.78, 1.50) | 1.13 (0.80, 1.58) |
| Atrialfibrillation/flutter | 0-3 months | 314,969 | 345 | Ref | Ref |
|  | 4-12 months | 137,770 | 172 | 1.10 (0.92, 1.32) | 1.08 (0.89, 1.30) |
|  | More than 12 months | 62,005 | 81 | 1.01 (0.79, 1.29) | 0.96 (0.74, 1.24) |
| Atherosclerosis | 0-3 months | 316,246 | 102 | Ref | Ref |
|  | 4-12 months | 138,373 | 43 | 0.92 (0.64, 1.31) | 0.91 (0.63, 1.32) |
|  | More than 12 months | 62,284 | 21 | 0.77 (0.48, 1.24) | 0.79 (0.49, 1.29) |
| Other cardiovascular diseases | 0-3 months | 304,168 | 2,447 | Ref | Ref |
|  | 4-12 months | 132,639 | 1,187 | 1.09 (1.02, 1.17) | 1.07 (0.99, 1.15) |
|  | More than 12 months | 59,507 | 544 | 1.03 (0.94, 1.13) | 1.02 (0.93, 1.13) |

^a^ Adjusted for age, education, smoking status, body-mass index, diabetes mellitus and parity.

Appendix 13. Sensitivity analysis of the relationship between time-to-pregnancy and risk of overall cardiovascular disease reclassifying a percentage of individuals with prolonged time-to-pregnancy to be in the reference category of 3 months or less

Adjusted for age, education, smoking status, body-mass index, diabetes mellitus and parity
